# Experimental Investigation on the Energy and Exergy Efficiency of the Vacuum Membrane Distillation System with Its Various Configurations

**DOI:** 10.3390/membranes14020054

**Published:** 2024-02-13

**Authors:** Abdullah Najib, Turki Mana, Emad Ali, Hany Al-Ansary, Fahad Awjah Almehmadi, Mansour Alhoshan

**Affiliations:** 1 Mechanical Engineering Department, King Saud University, P.O. Box 800, Riyadh 11421, Saudi Arabia; 2 Chemical Engineering Department, King Saud University, P.O. Box 800, Riyadh 11421, Saudi Arabia; turki.mana@gmail.com (T.M.); amkamal@ksu.edu.sa (E.A.); 3 Department of Applied Mechanical Engineering, College of Applied Engineering, Muzahimiyah Branch, King Saud University, P.O. Box 800, Riyadh 11421, Saudi Arabia; falmehmadi@ksu.edu.sa

**Keywords:** vacuum membrane distillation, hybrid configuration, performance indicators, vacuum pressure, exergy destruction percentage

## Abstract

This paper addresses a retrofitting vacuum membrane distillation (VMD) setup to reduce the accumulated pressure inside the permeated side. This modification is necessary to extend the operation of the VMD to extreme operation conditions of higher hot water temperatures. This modification, denoted as a hybrid configuration, proposes the injection of a cold water stream into the VMD cell without mixing it with the permeate. Energy and exergy efficiency analyses were performed to assess the effectiveness of the hybrid configuration. The performance of the modified system indicated an improvement in terms of permeate flux (J), the gain output ratio (GOR), and the utilitarian exergetic efficiency (η_ex,u_), which reach up to two and three times that of the base configuration of the VMD system. However, the exergetic efficiency (η_ex_) of the hybrid system showed marginal improvement compared to the base case over the tested range of hot water temperatures. This is because the enhanced vapor production is penalized by excess energy consumption. Moreover, the highest exergy destruction percentages occurred in the operational components (e.g., heater and chillers) which fall in the range of 19.0–68.9%. The exergy destruction percentage in the original components (e.g., the VMD cell and condenser) did not exceed 8.3%. Furthermore, this study indicated that the hybrid configuration requires additional tuning and optimization to perform efficiently over wide operating conditions.

## 1. Introduction

One of the most encouraging techniques for producing high-quality distillate water is desalination using membrane distillation (MD) technology. This technology is based on the combination of phase change and membrane filtration in the same module [1,2,3]. This technique uses thermal energy to heat up the saline water and cooling energy to cool down the permeate side in order to create a vapor pressure difference between the two sides of the hydrophobic membrane [1,4,5]. The amount of vapor produced that migrates through the hydrophobic membrane mainly depends on the extent of the vapor pressure difference and membrane characteristics. The main characteristics of the hydrophobic membrane that affect mass transfer are polymer types, porosity, pore size, tortuosity, diffusion area, and thickness [5].

Recently, practitioners and researchers have been studying different aspects of improving the performance of desalination systems using MD technology and its various configurations, especially in terms of increasing distillate production and/or reducing energy utilization, both thermal and electrical [6,7,8]. Several fruitful attempts dealing with linking MD systems to renewable energy sources (e.g., solar energy, geothermal energy, and waste energy) have been reported. For example, Guillen et al. [9] experimentally studied the performance of integrated air-gap membrane distillation (AGMD) with solar energy under specific operating conditions. They reported that the maximum permeate mass flux (J), performance ratio (PR), and specific thermal energy consumption (STEC) were in the range of 7 L·m^−2^·h, 0.79, and 810 kWh·m^−3^, respectively. Bouguecha et al. [10] verified the performance of direct contact membrane distillation (DCMD) combined with solar thermal collectors. The performance of DCMD was estimated under two modes: with a heat recovery system (arrangement B) and without HRD (arrangement A). The distillate water produced was 3.31 L·h^−1^ for arrangement A and 4.59 L·h^−1^ for arrangement B. They also found that the minimum STEC was 2342 kW·m^−3^ for arrangement A and 1609 kW·m^−3^ for arrangement B. Their study confirmed that the use of HRD has a positive impact on reducing the energy consumption. Najib et al. [11] experimentally investigated the effectiveness of a solar desalination system using the MD process. The study focused on evaluating the performance of a solar desalination system under the influence of weather conditions in Riyadh. The optimum performance indicators of their study, namely J, recovery ratio (R), and gain output ratio (GOR), were 12.2 kg·m^−2^·h, 36.8%, and 4.25, respectively. Another useful effort that dealt with using thermal energy recycling (e.g., multiple effects and joining them to a heat pump system) was also reported. Najib et al. [12] theoretically investigated the performance of the multiple effects vacuum membrane distillation (V-MEMD) module using energy and exergy analysis. The study focused on examining the optimal performance indicators over a wide range of operating conditions. They found the optimum performance indicator values of J, R, GOR, and STEC were 17.2 kg·m^−2^·h, 47.6%, 5.05, and 166 kWh·m^−3^, respectively. The results were considered to be extremely high due to the increased feed water temperature required by the system.

Currently, researchers are focusing on the internal modifications of MD and its various configurations [13,14,15] to enhance the mass transfer through the hydrophobic membrane. Vacuum membrane distillation is one of the attractive generations of MD. VMD applies a vacuum pressure at the permeating side using a vacuum pump. The idea is to enhance the driving force and, hence, the mass transfer. VMD is known to deliver the highest water flux due to its negligible conductive heat loss. Dong et al. [16] developed three open-source simulators using the Mathlab^®^ GUI platform to predict the performance of direct contact membrane distillation (DCMD), submerged vacuum membrane distillation (S-VMD), and cross-flow vacuum membrane distillation (X-VMD). Their simulators can predict large-scale MD performance. The study revealed that the heat lost in the radial direction was limited in the S-VMD configuration. Noticeable heat loss in the radial direction of the X-VMD was higher than that found in the S-VMD configuration due to the large, full-size aspect ratio. Conversely, the DCMD configuration was worse due to the conductive heat loss between the hot and cold water streams. VMD is highly thermally efficient because it eliminates the conduction heat loss of the membrane wall due to vacuum effects. Moreover, mass transfer resistance is decreased via deaeration. Ibrahim and Alshalhy [17] developed a mathematical model based on the fundamentals and governing equations of VMD configuration to predict the effect of mass and heat transfer on the permeate mass flux in a shell-and-hollow-fibers module. The model considered a combination of Kundsen diffusion and viscous flow mechanisms for vapor migration across the membrane during distillation. The conductive heat through the membrane during distillation was overcome due to the application of vacuum pressure on the permeate side. They found that the membrane characteristics (i.e., porosity, tortuosity, membrane types, thickness, and pore size) have a significant impact on the vapor mass transfer. In addition, their study described the influence of operating conditions (i.e., vacuum pressure, feed temperature, velocity, and feed salinity) and module properties (i.e., module length and packing density). Although many studies have revealed the preference of the VMD configuration over other MD configurations, unfortunately, this configuration still faces the limited capacity of its vacuum pump, especially when operating at extreme conditions of high feed temperature and/or feed flow rate. There are several studies that confirm a decline in the vacuum pressure level inside the permeate compartment as a result of increasing the hot water temperature, as reported in previous works [18,19]. This led to an increase in the absolute pressure within the permeate side of the VMD configuration, which was the main cause of the degradation of the mass flux. One way to overcome such ab issue is to use a vacuum pump with proper capacity. However, for large-scale application, the limitation may rise again under other extreme conditions. Therefore, this paper proposes another remedy involving the injection of cold water to the permeate side of the VMD system. Consequently, this work aims to experimentally investigate the effectiveness of such retrofitting on VMD performance. Through this study, the modified system is called the hybrid configuration. The hybrid configuration performance was assessed by energetic and exergetic analyses under specific operating conditions. The results are expressed in terms of permeate mass flux, gain output ratio, exergetic efficiency, utilitarian exergetic efficiency, and exergy destruction percentage. The outcome of this modification and its analysis may help in implementing VMD systems under wide operating conditions.

## 2. Facility Description

**Figure 1:**
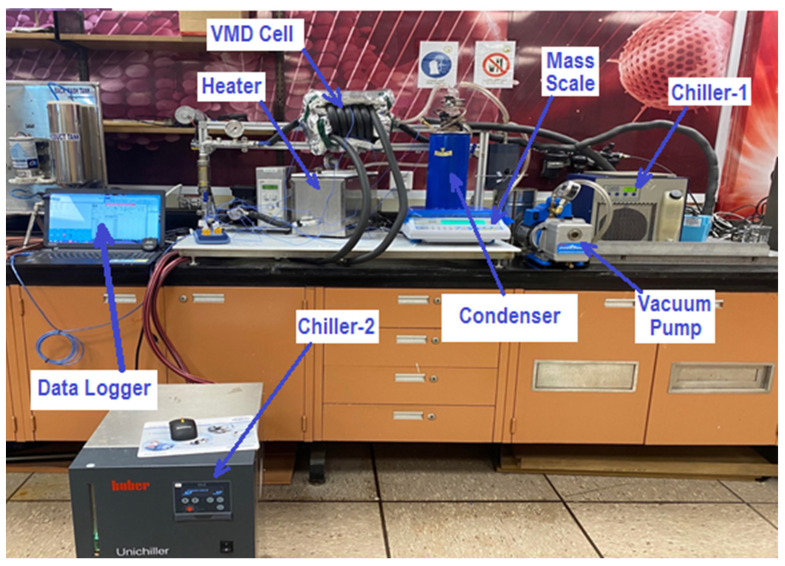
Photograph of the facility with its components.

**Figure 2:**
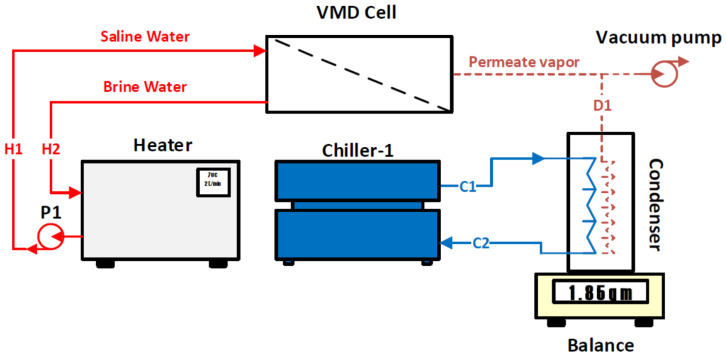
Base configuration of the VMD system.

The investigated VMD system was designed and implemented at the King Abdullah Institute for Nanotechnology (KAIN/NAN) in the kingdom of Saudi Arabia (KSA). Figure 1 shows a photograph of the developed system. The VMD setup consists of six major components: a heater, a VMD cell, a vacuum pump, Chiller-1, Chiller-2, and a condenser. The heater was manufactured by Galli company (Milan, Italy), and it consists of a stainless-steel heating tank with a capacity of 4 L, which heats a synthetic salt solution (i.e., saline water) and sends it to the VMD cell via a pump (P1) at state H1, as shown in Figure 2. The hot water flow ranges from 2 L/min to 6 L/min. The feed temperature is controlled by the electrical heater which can regulate the temperature to lie within 40–70 °C. The separation process in the VMD cell depends on the pressure gradient across the hydrophobic membrane. The pressure gradient is generated by applying vacuum pressure to the permeate side. The vapor extracted from the hot side through the membrane transfers to the condenser at state D1. There are two cold water streams that are used in the permeate side and generated using two separate chillers. The first chiller was manufactured by Oasis company (Columbus, OH, USA) and has an input power of 0.16 kW; it is used to condensate the extracted permeate vapor. The second chiller was manufactured by Huber company (Offenburg, Germany) and has an input power of 0.6 kW; it is used only in the hybrid configuration. The purpose of the second chiller is to cool down the permeate side’s temperature in order to reduce its internal pressure. The brine discharged from the VMD cell ends up in a heating tank at state H2. To maintain continuous operation, the reject brine is heated again in the heater and recycled back to the cell. The feed flow rate and temperature are measured by a regulator (ABB) and thermocouple data logger (Pico-TC-08), respectively. The vacuum pressures inside the permeate side are measured by a vacuum pressure gauge. The collected distillate is measured by an A&D Weighing lab balance (Tokyo, Japan) and its salinity is measured by a Delta OHM pH/conductivity meter (Padua, Italy).

**Figure 3:**
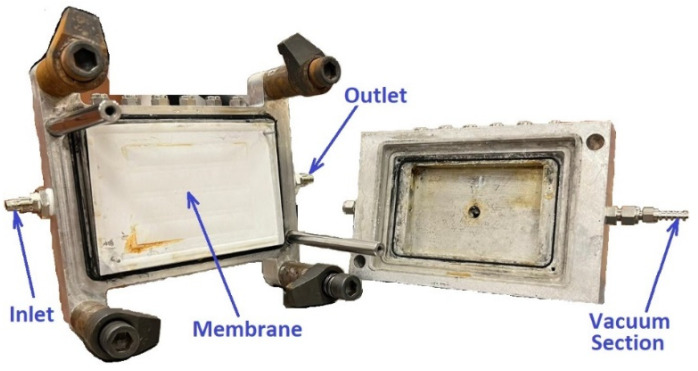
Photograph of the VMD cell.

The VMD cell’s outer frame is made of stainless-steel material and equipped with an inlet and outlet for a saline water stream under applied vacuum pressure, as shown in Figure 3. The VMD cell was fastened on a steel structure and placed in the center of the VMD system. The VMD cell is thermally insulated to minimize heat losses to the surrounding environment. The characteristics and specifications of the commercial membrane used in this work are listed in Table 1.

## 3. Hybrid Configuration of the VMD System

**Figure 4:**
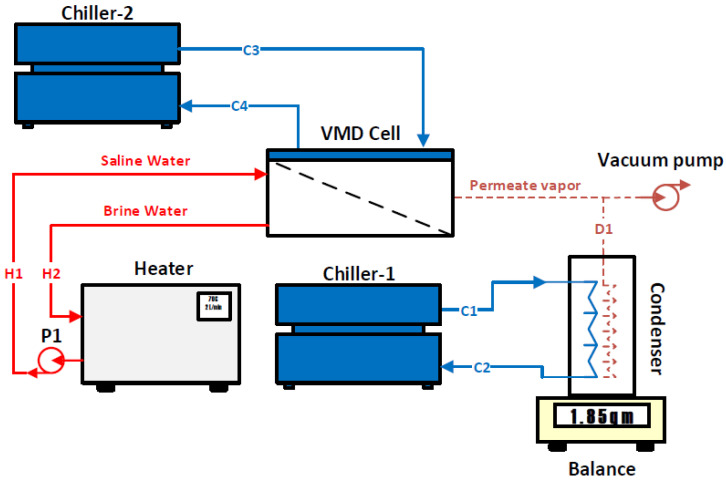
Hybrid configuration of the VMD system.

The basic operation of the VMD module is based on raising the hot water temperature by the heater to the desired temperature. On the other side, the vacuum pressure is applied to the permeate side in order to create a vapor pressure difference between the two interfaces of the hydrophobic membrane, as reported by previous research works [1,15]. The pressure difference forces the vapor to flow from the hot side, diffuse through the hydrophobic membrane to the permeate side, and end up at the condenser. In the condenser, the vapor is condensed by means of a cold water stream circulated by the first chiller. This configuration is called the base configuration of the VMD system. During several experiments conducted using this configuration, high-pressure entrapment within the permeate side was observed. This situation hindered the vapor flow across the hydrophobic membrane because the vacuum pump was unable to further reduce the pressure, as shown in Figure 2. A novel remedy to this problem is to eliminate the pressure accumulation inside the permeate side by injecting a cold water stream into the VMD cell without mixing it with the permeate vapor. This configuration is called the hybrid configuration, as shown in Figure 4. Indeed, this proposed idea had a significant impact on improving the permeate mass flux across the hydrophobic membrane as is discussed in detail in the following sections.

## 4. Methodology

A schematic diagram of the VMD system is shown in Figure 4. The system consists of a VMD cell, a heater, two chillers, a condenser, and pumps (i.e., vacuum pump and hot water pump (P1)). The conservation equations were applied for each component of the VMD system except for the pumps. The following typical assumptions are considered:

1. Steady state;
2. Both kinetic and potential energies are ignored;
3. Neglecting fluid leakage of the VMD system’s components;
4. Consider the coefficient of performance (COP) of the two chillers to be equal to 3;
5. Consider that the vapor extracted from the VMD cell is in vapor phase;
6. Complete condensation is assumed in the condenser compartment.

### 4.1. Mass, Energy, and Exergy Balances

**Figure 5:**
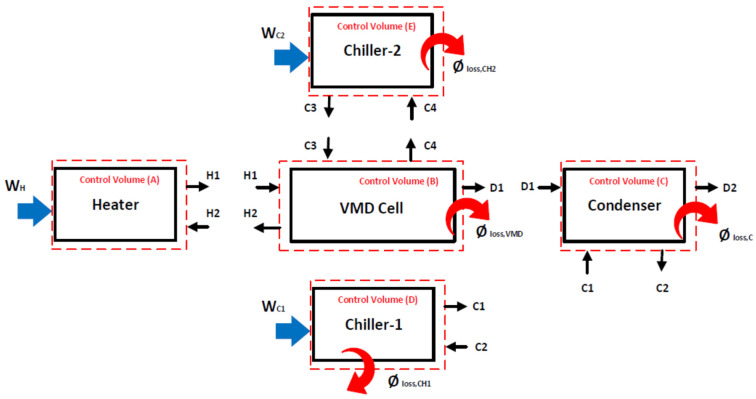
Control volumes for main components of the VMD system.

Conservation equations were applied to each component of the VMD system using control volumes in order to predict the unmeasured variables such as heat loss and work performed on some components. These control volumes are shown in Figure 5. The outcome of the conservation equations help in estimating the performance indicators to be used in evaluating the VMD system [12,20].

As specified in Figure 5, the conservation equations can be written for each control volume as fully described in the following section. Control volume (A) is designated for the heater, which consists of a stainless-steel heating tank with a capacity of 4 L. This unit heats up the saline water to the desired temperature and pushes it into the VMD cell via a pump (P1), as shown in Figure 2 and Figure 4. The mass, energy, and exergy balances can be written as follows:

(1) $${\dot{m}}_{H1}-{\dot{m}}_{H2}=\frac{dm}{dt}$$

(2) $$W_{H}+{\dot{m}}_{H2}h_{H2}-{\dot{m}}_{H1}h_{H1}=\frac{dE}{dt}$$

(3) $$W_{H}+{\dot{m}}_{H2}\varphi_{H2}-{\dot{m}}_{H1}\varphi_{H1}-\Psi_{des,H}=\frac{d\Psi }{dt}$$

Control volume (B) is devoted for the VMD cell which includes a hot water stream and a cold water stream. The latter is neglected when considering the base configuration (i.e., the absence of C3 and C4), as described in Figure 2. Conservation equations can be expressed through this control volume as follows:

(4) $${\dot{m}}_{H1}+{\dot{m}}_{C3}={\dot{m}}_{H2}+{\dot{m}}_{C4}+{\dot{m}}_{D1}$$

(5) $$\left[\begin{array}{c} {\dot{m}}_{H1}h_{H1}+{\dot{m}}_{C3}h_{C3}+\emptyset_{gain,VMD}={\dot{m}}_{H2}h_{H2}\\ +{\dot{m}}_{C4}h_{C4}+{\dot{m}}_{D1}h_{D1,g}+\emptyset_{loss,VMD} \end{array}\right]$$

(6) $$\left[\begin{array}{c} {\dot{m}}_{H1}\varphi_{H1}+{\dot{m}}_{C3}\varphi_{C3}-{\dot{m}}_{H2}\varphi_{H2}\\ -{\dot{m}}_{C4}\varphi_{C4}-{\dot{m}}_{D1}\varphi_{D1,g}+\emptyset_{gain,VMD}\left[1-\frac{T_{i}}{T_{H,avg}}\right]\\ -\emptyset_{loss,VMD}\left[1-\frac{T_{i}}{T_{H,avg}}\right]=\Psi_{des,VMD} \end{array}\right]$$

The minimum work applied on the VMD cell is defined as the difference between the exergies of the outlet and inlet of the hot water stream; it can be expressed as follows:

(7) $$W_{min}={\dot{m}}_{H2}\varphi_{H2}+{\dot{m}}_{D1}\varphi_{D1}-{\dot{m}}_{H1}\varphi_{H1}$$

where $\dot{m}$, h, φ, $\emptyset_{loss,VMD}$, and $W_{min}$ denote mass, enthalpy, exergy flow, heat lost or heat gain from the VMD cell, and the minimum work required to separate the vapor from the saline water, respectively. H1 and H2 refer to the inlet and outlet’s hot water, respectively. C3 and C4 refer to the inlet and outlet’s cold water, respectively. D1 indicates the vapor produced.

Control volume (C) is designated for the condenser, through which all vapor produced is completely condensed; the following mass, energy, and exergy balances can be expressed:

(8) $${\dot{m}}_{D1}+{\dot{m}}_{C1}={\dot{m}}_{D2}+{\dot{m}}_{C2}$$

(9) $${\dot{m}}_{D1}h_{D1,g}+{\dot{m}}_{C1}h_{C1}+\emptyset_{gain,C}={\dot{m}}_{D2}h_{D1,f}+{\dot{m}}_{C2}h_{C2}+\emptyset_{loss,C}$$

(10) $$\begin{array}{c} {\dot{m}}_{D1}\varphi_{D1,g}+{\dot{m}}_{C1}\varphi_{C1}-{\dot{m}}_{D2}\varphi_{D1,f}-{\dot{m}}_{C2}\varphi_{C2}+\emptyset_{gain,C}\left[1-\frac{T_{i}}{T_{D1}}\right]\\ -\emptyset_{loss,C}\left[1-\frac{T_{i}}{T_{D1}}\right]{=\Psi }_{des,C} \end{array}$$

Control volume (D) comprises the first chiller. The function of the first chiller is absorbing the heat rejected from the permeate stream; the following mass, energy, and exergy balances can be expressed:

(11) $${\dot{m}}_{C1}={\dot{m}}_{C2}$$

(12) $$W_{CH1}+{\dot{m}}_{C2}h_{C2}-{\dot{m}}_{C1}h_{C1}=\emptyset_{loss,CH1}$$

(13) $$W_{CH1}+{\dot{m}}_{C2}\varphi_{C2}-{\dot{m}}_{C1}\varphi_{C1}-\emptyset_{loss,CH1}\left[1-\frac{T_{i}}{T_{CH1}}\right]=\Psi_{des,CH1}$$

Control volume (E) comprising the second chiller is only considered in the hybrid configuration. The second chiller aims to cool down the permeate side of the VMD cell and, hence, allow the vacuum pressure to increase; the following mass, energy, and exergy balances can be expressed:

(14) $${\dot{m}}_{C3}={\dot{m}}_{C4}$$

(15) $$W_{CH2}+{\dot{m}}_{C4}h_{C4}-{\dot{m}}_{C3}h_{C3}=\emptyset_{loss,CH2}$$

(16) $$W_{CH2}+{\dot{m}}_{C4}\varphi_{C4}-{\dot{m}}_{C3}\varphi_{C3}-\emptyset_{loss,CH2}\left[1-\frac{T_{i}}{T_{CH2}}\right]=\Psi_{des,CH2}$$

It is possible to predict the work supplied to the chiller by knowing the coefficient of performance (COP) of the chiller, which is estimated from its technical specifications provided by the vendor

(17) $$W_{CH1}=\frac{{\dot{m}}_{C2}h_{C2}-{\dot{m}}_{C1}h_{C1}}{COP}$$

And

(18) $$W_{CH2}=\frac{{\dot{m}}_{C4}h_{C4}-{\dot{m}}_{C3}h_{C3}}{COP}$$

where $W_{CH1}$, $W_{CH2}$, and $\Psi_{des}$ denote the work supplied to the first and second chiller and exergy destruction, respectively.

### 4.2. Performance Evaluation

Several performance indicators are used for evaluating the performance of desalination systems using VMD, which are classified into performance indicators related to productivity, energy, and exergy. The following section represents these performance indicators.

One critical performance indicator is the permeate mass flux (J), which is used to assess the efficiency of the hydrophobic membrane by estimating the amount of vapor diffusing through the membrane per square meter in a specified time; it can be expressed as follows [11,12]:

(19) $$J=\frac{{\dot{m}}_{D1}}{A}$$

Another important performance indicator is the gain output ratio (GOR), which is widely used in the assessment of thermal energy efficiency of desalination systems. It can be determined [21] as follows:

(20) $$GOR=\frac{{\dot{m}}_{D1}h_{D1,fg}}{W_{H}}$$

Exergetic efficiency is used in this study to assess the performance of the separation process; it can be defined as a ratio of the minimum work required to the heat supplied to the VMD cell by the hot water stream as follows [12,20]:

(21) $$\%\eta_{ex}=\frac{W_{min}}{{\dot{m}}_{H1}\varphi_{H1}}$$

Utilitarian exergetic efficiency is also used to determine the real exergy required to condensate the vapor produced from the VMD cell to the exergy that drives the entire system; it can be expressed as follows [20]:

(22) $$\%\eta_{ex,u}=\frac{{\dot{m}}_{D1}h_{D1,fg}\left[1-\frac{T_{i}}{T_{D1}}\right]}{W_{H}+W_{CH1}+W_{CH2}}$$

Exergy destruction percentage is one of the critical performance indicators used to detect the maximum exergy destruction in a VMD system’s components; it can be defined as the ratio of exergy destruction occurring in a specific component to the total exergy destruction of the VMD system [22]:

(23) $$\%\Psi_{des,i}=\left[\frac{\Psi_{des,i}}{\sum \Psi_{des,i}}\right]\times 100$$

It should be noted that the dead state for the exergy calculations is characterized by 9.1 °C, 101.3 kPa, and 5 ppm.

## 5. Results and Discussion

A comprehensive experimental study of the hybrid configuration of the VMD system under specified operating conditions was conducted. The study relies on the energy and exergy efficiency analysis in evaluating the performance indicators of the hybrid configuration compared to the base configuration of the VMD system. The goal is to enhance the efficiency of the VMD system through optimal leveraging of the thermal energy inside the VMD cell. The hot feed is a synthetic salt solution with an approximate salinity of 65,000 ppm (i.e., 65 g/kg), and its thermodynamics properties are estimated considering the solution as a real mixture [23,24]. The cold streams’ conditions for both chillers were kept constant. Obviously, ethylene glycol was used in the second chiller as a cooling stream for the VMD cell and its thermodynamics properties were treated as those of a brine [25,26]. The following sections present the outcomes of the analysis.

### 5.1. Effect of Feed Flow and Temperature on the VMD System

**Figure 6:**
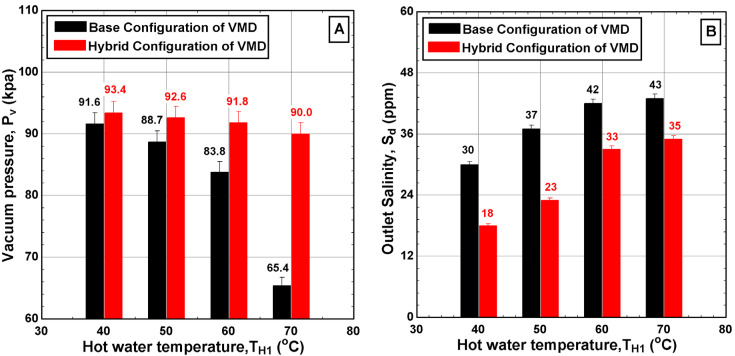
Effect of the hot water temperature T_H1_ on (**A**) vacuum pressure into the VMD cell and (**B**) outlet salinity of produced water for different configurations (${\dot{v}}_{H1}$ = 2 L/min, $C_{H1}$ = 65,000 ppm, ${\dot{v}}_{C1}$ = 2 L/min, $T_{C1}$ = 8.6–11.4 °C, ${\dot{v}}_{C3}$ = 4.5 L/min, and $T_{C3}$ = 10.7–23.1 °C).

In this section, we illustrate the effect of feed flow rate and feed temperature on the behavior of both the base and hybrid configurations. Figure 6 shows the variation in process variables such as the vacuum pressure and outlet salinity of the distillate water over a range of hot water temperatures. Increasing the hot water temperature led to a significant decrease in the vacuum pressure for the two configurations but at different rates, which complies with the vacuum pressure behaviors reported in previous works [18,19]. It was evident that the hybrid configuration approximately maintained the vacuum pressure at high values over the entire range of tested temperatures with an average value of 92 kPa. Moreover, the maximum enhancement percentage of the vacuum pressure was registered at the highest hot feed temperature (T_H1_~70 °C) which reaches 38% over the base configuration, as shown in Figure 6A. In addition, the maximum reduction percentage of the outlet salinity compared to that of the base configuration can reach up to 40% at the lowest operating temperature, as shown in Figure 6B. Of course, the reduction in product salinity is the outcome of maintaining high vacuum pressure.

**Figure 7:**
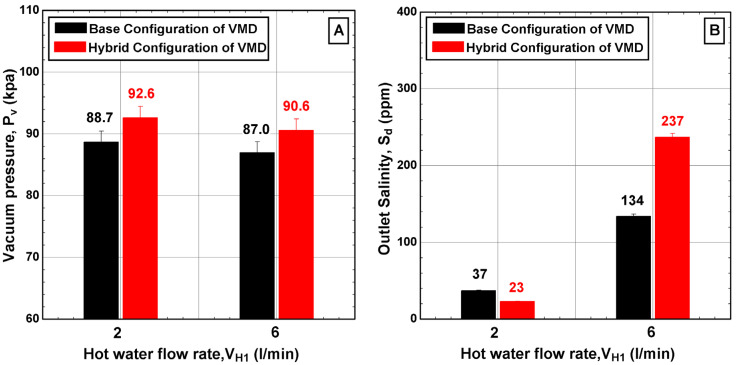
Effect of the hot water flow rate V_H1_ on (**A**) vacuum pressure into the VMD cell and (**B**) outlet salinity of produced water for different configurations ($T_{H1}$ = 50 °C, $C_{H1}$ = 65,000 ppm, ${\dot{v}}_{C1}$ = 2 L/min, $T_{C1}$ = 8.9–10.3 °C, ${\dot{v}}_{C3}$ = 4.5 L/min, and $T_{C3}$ = 12.9 °C).

Figure 7 illustrates the variations in the process variables at two values of hot feed flow rate, specifically 2 L/min and 6 L/min. It was observed that increasing the hot water flow rate had a minimal effect on the vacuum pressure, as only a 5% enhancement in the vacuum pressure of the hybrid configuration compared to the base case was obtained, as depicted in Figure 7A. However, a surprising finding was the impact of flow rate on the outlet salinity of the distillate water, which soared to 237 ppm at the highest hot water flow rate of 6 L/min. Further investigation revealed that the filtration process failed when operating at hot water temperatures above 50 °C and at the highest flow rate of 6 L/min. This failure was mainly attributed to the experimental setup being forced to operate beyond its design capacity. Additionally, the increasing salinity in the distillate could be an indication of pore wetting due to the enlarged hydrostatic pressure induced by elevated flow rate. Other possible reasons include the membrane infrastructure, increased dredging of salt particles during the increase in the evaporation process, and the internal reaction between hot water and the membrane. These findings emphasize the importance of upscaling procedure. Even with the enlargement of lateral components, challenges may persist in large-scale applications. Therefore, additional techniques or modifications are necessary to ensure that the existing process can operate effectively over a wide range of operating conditions.

### 5.2. Energy and Exergy Flow Diagrams

**Figure 8:**
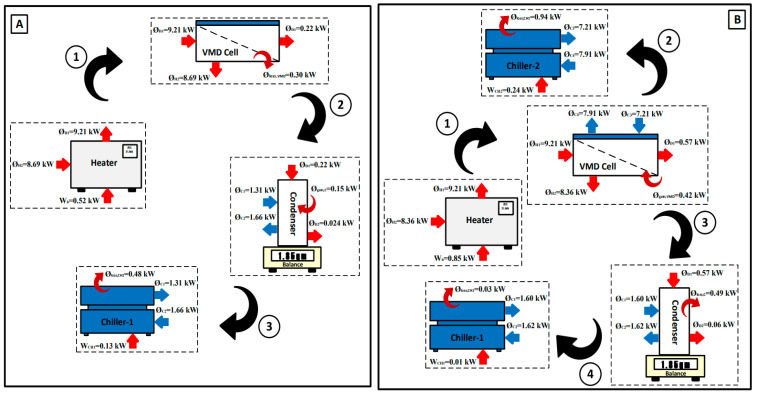
Energy diagram of the VMD system: (**A**) base configuration and (**B**) hybrid configuration.

This section aims at understanding and evaluating the energy and exergy and their reflection on the VMD system and its components for both the base and hybrid configurations. This step enables determining the performance of each component independently or as an integrated part of the entire VMD system by using the measured data and the conservation equations described in Section 4. First, we consider the base case. Table 2 shows the experimentally measured data and their thermodynamics properties for different locations of the base configuration under specific operating conditions in the absence of a second chiller. Exergy flow rates were treated using the approach of Sharqawy and exergy flow rate values relative to the Sharqawy model which are always positive [23,24]. The minimum value of the exergy flow rate was recorded at state C1, which appeared to be almost zero when the absolute pressure was slightly higher than the dead-state pressure. The maximum value of the exergy flow rate was recorded at state D1 due to the vapor phase and its high temperature regardless of the effect of the absolute pressure, which reached to 36 kPa. Figure 8A demonstrates the resulting energy values. As thermal energy is supplied via the heater system to the VMD cell, water vapor is generated and diffuses through the hydrophobic membrane due to the vacuum pressure applied at the other side of the membrane. However, during the experimental test, high pressure builds up in the permeate side which decreases the productivity considerably until it reaches a low value of 0.005 kg/min. In addition, the heat lost to the surroundings surges, incurring an additional heat load of 0.30 kW out of 0.52 kW. In the condenser compartment, the extracted vapor is condensed via the first chiller which also suffers from heat gain from the surroundings; this raises the cooling load on the first chiller to 0.15 kW out of 0.35 kW, as shown in Figure 8A. Secondly, Table 3 shows the experimentally measured data and their thermodynamic properties at different locations of the hybrid configuration under specific operating conditions with the existence of a second chiller. The minimum value of the exergy flow rate was recorded at state C1 which did not exceed 0.03 kJ/kg when the temperature and the absolute pressure were slightly higher than the dead-state conditions. The maximum value of the exergy flow rate was also recorded at state(D1 for the same reasons mentioned above, with an absolute pressure that may reach up to 11.3 kPa. Figure 8B displays the resulting energy values. The main purpose of incorporating the second chiller into the VMD cell is to reduce the pressure trapped in the permeate side to allow additional vapor to flow through the hydrophobic membrane (i.e., more productivity). Indeed, the productivity becomes approximately three times greater than that of the base configuration. Unfortunately, the VMD cell gained heat from the surroundings due to the lowered temperature of the permeate side. As a result, the cooling load on the second chiller grows to 0.42 kW out of 0.70 kW. On the other hand, there was a significant decrease in the cooling load on the first chiller which might be attributed to variation in the vapor phase (i.e., quality of vapor), as shown in Figure 8B.

**Figure 9:**
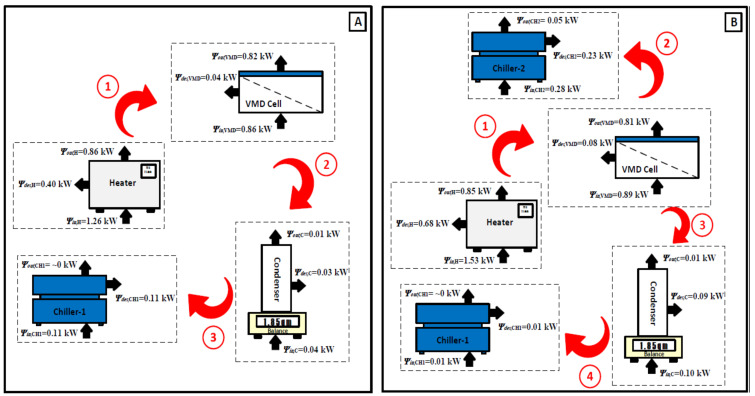
Exergy diagram of the VMD system: (**A**) base configuration and (**B**) hybrid configuration.

Figure 9 shows the exergy diagram for each component of the VMD system. The maximum exergy destruction values were registered in the heater system; values for base and hybrid configurations can reach up to 0.40 kW and 0.68 kW, respectively. The reason for increased exergy destruction in the hybrid configuration is the increased temperature of the saline water in the VMD cell, which incurred additional load from the heater. Conversely, the exergy destruction values in the VMD cell and the condenser were marginal for both configurations which amounted to 0.04–0.08 kW and 0.03–0.09 kW. The hybrid configuration exhibited slightly higher exergy destruction due to the increase in heat interaction with the surroundings. Finally, the exergy destruction values of the chillers for both configurations owned a larger proportion of the inlet exergies. This might be ascribed to the fact that the heat rejected from chillers was not fully harnessed.

### 5.3. Performance Evaluation

**Figure 10:**
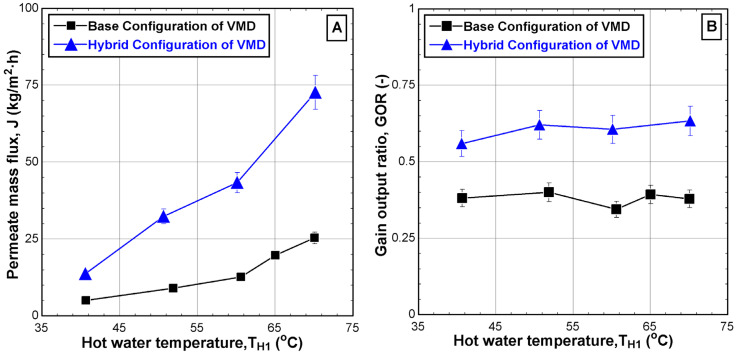
Effect of the hot water temperature T_H1_ on (**A**) permeate mass flux and (**B**) gain output ratio for different configurations (${\dot{v}}_{H1}$ = 2 L/min, $C_{H1}$= 65,000 ppm, ${\dot{v}}_{C1}$ = 2 L/min, $T_{C1}$ = 8.6–11.4 °C, ${\dot{v}}_{C3}$ = 4.5 L/min, and $T_{C3}$ = 10.7–23.1 °C).

Performance evaluation in this study is based on the performance indicators directly related to productivity, energy, and exergy, as described in Section 4.2. Figure 10 shows the effect of the hot feed temperature on the permeate mass flux (J) and the gain output ratio (GOR). The permeate mass flux increased significantly when the hot water temperature increased from around 40 °C to 70 °C as shown in Figure 10A. The mass flux is increased for both configurations but at different slopes with the maximum value for the base and hybrid configurations reaching 25.3 kg/m^2^·h and 72.6 kg/m^2^·h, respectively. The impact of feed temperature on the hybrid configuration was evident compared to the base configuration, as the permeate mass flux was nearly threefold greater than the base case, as shown in Figure 10A. Additionally, the performance indicator (GOR) is directly linked to the permeate mass flux but is also related to the thermal energy supplied by the heater. Indeed, the GOR for the base and hybrid configuration is found to be almost invariant with feed temperature. The average values of GOR are around 0.38 and 0.61 for base and hybrid structures, respectively, over the hot water temperature range. The stability of the GOR might be attributed to an increase in the supplied thermal energy at the same rate as the increase in vapor extracted from the VMD cell. Moreover, the impact of the hybrid configuration was clearly visible as the GOR can be twice that of the base configuration, as shown in Figure 10B. The enhancement in J and the GOR of the hybrid system is ascribed to the maximization of the pressure drop across the hydrophobic membrane when the second chiller is incorporated. This issue is also confirmed in previous works [11,12,27].

**Figure 11:**
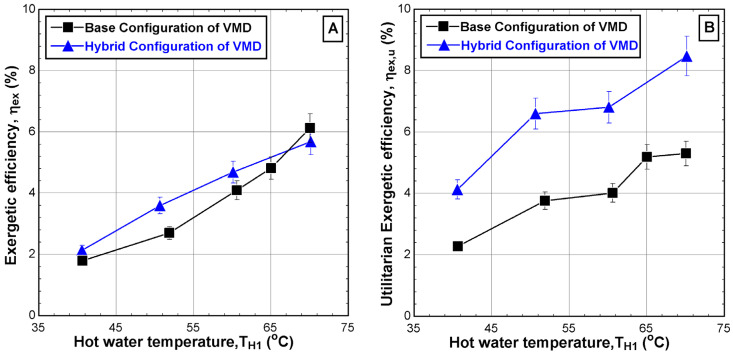
Effect of the hot water temperature T_H1_ on (**A**) exergetic efficiency and (**B**) utilitarian exergetic efficiency for different configurations (${\dot{v}}_{H1}$ = 2 L/min, $C_{H1}$ = 65,000 ppm, ${\dot{v}}_{C1}$ = 2 L/min, $T_{C1}$ = 9.1–11.2 °C, ${\dot{v}}_{C3}$ = 4.5 L/min, and $T_{C3}$ = 10.7–23.1 °C).

Figure 11 shows the effect of the hot feed temperature on the two types of exergetic efficiencies, namely the exergetic efficiency (η_ex_), which is related to the quality of the separation process within the VMD cell, and the utilitarian exergetic efficiency (η_ex,u_), which is related to the real exergy required to condense the vapor. Figure 11A depicts the improvement of the exergetic efficiency with the hot feed temperature for both configurations with an overall enhancement of 6% for both cases. However, the improvement of the performance of the hybrid system over the base case is minor. In fact, the enhanced production of the hybrid system is penalized by growth in the heat supply by the heater. The use of a second chiller and the elevated vapor production cools down the exit brine substantially. Note that the experiment is working in a closed-loop mode, i.e., the reject brine is heated in the heater and recycled into the VMD cell. Therefore, the heat supply surges to maintain the feed water at the desired temperature. In reality, the process operates in open-loop mode, i.e., continuous feed operation. In this case, the surging heat supply is avoided.

As described in Figure 11B, the utilitarian exergetic efficiency increased significantly when the hot feed temperature was increased from 40 °C to 70 °C for both configurations. The maximum increment reaches up to 5.3% for the base configuration and 8.5% for the hybrid configuration. Although additional electrical energy is consumed by the hybrid configuration due to the utilization of the second chiller, the corresponding utilitarian exergetic efficiency is superior to the base configuration which could be twice as much.

**Figure 12:**
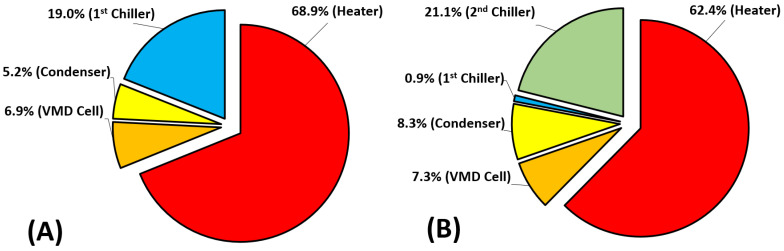
Exergy destruction percentage of the main components of the VMD system: (**A**) base configuration and (**B**) hybrid configuration (${\dot{v}}_{H1}$ = 2 L/min, $T_{H1}$ = ~70 °C $C_{H1}$ = 65,000 ppm, ${\dot{v}}_{C1}$ = 2 L/min, $T_{C1}$ = 9.1–11.2 °C, ${\dot{v}}_{C3}$ = 4.5 L/min, and $T_{C3}$ = 23.1 °C).

Figure 12 describes the exergy destruction in the major components of the VMD system for both configurations. Based on Table 2 and Table 3, the total exergy destruction of the base configuration ($\Psi_{des}=0.58\;kW$) is less than that of the hybrid configuration ($\Psi_{des}=1.09\;kW$). The results showed that the highest percentages of exergy destruction occurred in the operational systems, such as the heater and chillers. Apparently, the exergy destruction of the heater in the hybrid system is lower than that of the base case by 6.5%. Regarding the chillers, the combined exergy destruction of the first and second chillers in the hybrid system amounts to 22%, which is 3% higher than that in the base case, which indicates higher activity in the proposed configuration. Interestingly, an extremely greater amount of exergy is lost in the second chiller compared to the first chiller in the hybrid system. This may indicate that most of the cooling and probably condensation occurs in the second chiller to the extent that the first chiller can be excluded. This calls for optimization of the individual units to achieve optimal operation. In contrast, the results also showed that the lowest percentages of exergy destruction occurred in the original systems, such as the VMD cell and the condenser. The discrepancy percentages between the two configurations for these components did not exceed 0.4% and 3.1%.

Table 4 summaries the main performance indicators directly related to productivity, energy, and exergy, which are J, GOR, $\eta_{ex}$, and $\eta_{ex,u}$ under specific operating conditions. The table represents a comparison between previous works [12,20] and the present work under two configurations. The results of the present work indicated that the performance indicators related to productivity (e.g., J) were extremely high compared to those reported in Najib’s work [12]. This might be attributed to several reasons: (i) the temperature distribution along membrane area and (ii) energy dissipation through the multi-effect areas. In addition, the reported GOR, $\eta_{ex}$, and $\eta_{ex,u}$ engulf our findings, but the GOR values of the base configuration were slightly lower. This is due to the influence of the pressure trapped inside the permeate side of the VMD cell due to overheating. As a result, the accumulated pressure hindered mass flux and, subsequently, the separation process.

## 6. Conclusions

The pressure drop across the membrane plays an important role in vacuum membrane distillation technology. High-pressure entrapment in the permeate side leads to a counterforce that reduces the vapor flow, especially at high temperatures of the hot feed. One solution is augmenting the VMD system by a second chiller to cool the permeate side. The proposed modification (hybrid configuration) aims at improving the VMD performance, especially at high temperatures of the hot water (T_H1_~70 °C). A maximum improvement in the vacuum pressure of about 38% was obtained. Moreover, the salinity of the distillate reduced to around 35 ppm. Interestingly, the filtration process failed in the hybrid configuration at extreme feed flow rates (i.e., 6 L/min and 70 °C). This calls for further investigation to tune and/or redesign the system.

The results of energy and exergy analysis confirmed the superiority of the hybrid system. In fact, the production rate, GOR, and utilitarian exergy can be enhanced by two to three times over the base configuration. Unfortunately, the impact of the proposed system on the exergetic efficiency (η_ex_) was minor over the hot water temperature range. In fact, the improved vapor extraction was counteracted by the excess energy consumption by the heater. The increased heater supply was necessary to maintain the VMD feed at the desired temperature, bearing in mind that the recycled brine cools down considerably in the hybrid mode. It was also found for both configurations that the highest exergy destruction percentages occurred in the operational components (e.g., heater and chillers) and fell in the range of 19.0–68.9%. In conclusion, the preliminary analysis proved the success of the combined use of vacuum pumping and cooling of the permeate side to improve VMD performance in terms of production rate. However, further tuning and optimization of the overall system along with its components are necessary to expand the operability of the system.

## Figures and Tables

**Table 1:** Characteristics and specifications of the commercial membrane.

Character of Layer	Detail
Hydrophobic membrane	Polytetrafluoroethylene (PTFE)
Dimension	12 mm × 9 mm
Effective area of one effect	0.0108 m^2^
Membrane thickness	64–127 μm
Porosity	~75%
Tortuosity	~1.33
Mean pore size	~0.45 µm

**Table 2:** Thermodynamic properties for different locations of the base configuration.

State	Liquid	T (°C)	P_a_ (kPa)	m° (kg/min)	C (ppm)	h (kJ/kg)	s (kJ/kg·k)	φ (kJ/kg)
**H1**	Saline water	70.1	155.0	~2.046	65,000	270.0	0.87	25.29
**H2**	Saline water	66.4	134.0	~2.041	65,152	255.5	0.82	22.79
**D1**	Distillate water	69.7	36.0	~0.005	43	2626.0	7.76	437.10
**D2**	Distillate water	67.1	101.3	~0.005	43	280.9	0.92	22.06
**C1**	Distillate water	9.1	118.0	~2.050	5	38.3	0.14	~0.00
**C2**	Distillate water	11.6	107.2	~2.050	5	48.5	0.17	0.05

**Table 3:** Thermodynamic properties for different locations of the hybrid configuration.

State	Liquid	T (°C)	P_a_ (kPa)	m° (kg/min)	C (ppm)	h (kJ/kg)	s (kJ/kg·k)	φ (kJ/kg)
**H1**	Saline water	70.2	157.5	~2.046	65,000	270.3	0.87	25.35
**H2**	Saline water	63.8	133.5	~2.033	65,438	245.5	0.80	21.17
**D1**	Distillate water	70.0	11.3	~0.013	35	2626.0	7.75	439.7
**D2**	Distillate water	69.6	101.3	~0.013	35	291.3	0.95	23.88
**C1**	Distillate water	11.2	122.5	~2.050	5	46.8	0.17	~0.03
**C2**	Distillate water	11.3	110.5	~2.050	5	47.3	0.17	~0.04
**C3**	Ethylene glycol	23.1	132.5	~4.500	50% in water	96.2	0.34	0.86
**C4**	Ethylene glycol	28.4	110.0	~4.500	50% in water	105.6	0.37	1.37

**Table 4:** Comparison of the present results with those of [12,20].

Reference Number	Miladi et al. [20]	Najib et al. [12]	The Present Work	
			Base	Hybrid
Membrane distillation configuration	VMD	V-MEMD	VMD	VMD
Membrane distillation module	Hollow fiber	Flat sheet	Flat sheet	Flat sheet
Membrane area, A (m^2^)	N/A	5.12	0.0108	0.0108
Feed water type	liquid desiccant (LiCl)	Brackish water	a synthetic salt solution	a synthetic salt solution
Hot water temperature, $T_{H1}$ (°C)	~81	55–75	40–71	40–71
Hot flow rate, ${\dot{v}}_{H1}$ (L/h)	~2232 ^b^	840	120–360	120–360
Cold-side absolute pressure, $P_{a,v}$ (kpa)	7.0	11.5	9.7–35.9	7.9–11.3
Cold water temperature, $T_{C1}$ (°C)	29.0	20.0–45.0	9.1–11.2	9.1–23.1
Feed flow rate, ${\dot{v}}_{F}$ (L/h)	N/A	87–159	N/A	N/A
Distillate water, J (kg/m^2^·h)	N/A	0.6–17.2	5.0–25.3	13.6–72.6
Gain output ratio, GOR	2.20 ^b^	0.40–5.05	0.37–0.38	0.55–0.63
Exergetic efficiency, η_ex_ (%)	2.3–3.25	0–18.2	1.8–6.1	2.1–5.7
Utilitarian exergetic efficiency, η_ex,u_ (%)	9.96	2.5 ^b^–57.4 ^b^	2.2–5.3	4.1–8.5

N/A: not available; ^b^ values calculated from the reported data.

## Data Availability

The original contributions presented in the study are included in the article, further inquiries can be directed to the corresponding authors.

## Nomenclature

A	Membrane area, m^2^
C	Salinity, ppm
COP	Coefficient of performance
GOR	Gain output ratio
h	Enthalpy, kJ/kg
$h_{D1,g}$	Gas phase of the permeate flow, kJ/kg
$h_{D1,f}$	Liquid phase of the permeate flow, kJ/kg
$h_{D1,fg}$	Latent heat, kJ/kg
J	Permeate flux, kg/m^2^·h
m°	Mass flow rate, kg/s
P	Pressure, kpa
$P_{v}$	Vacuum pressure, kPa
$P_{a}$	Absolute pressure, kPa
T	Temperature, °C
$T_{i}$	Reference temperature, °C
$T_{CH1}$	First chiller condenser’s temperature, °C
$T_{CH2}$	Second chiller condenser’s temperature, °C
$W_{H}$	Electrical work supplied on the heater, W
$W_{CH1}$	Work driven on the first chiller, W
$W_{CH2}$	Work driven on the second chiller, W
$W_{min}$	Minimum work, W
v	Volume flow rate, L/h
Greek	
η_ex_	Exergetic efficiency, %
η_ex,u_	Utilitarian exergetic efficiency, %
$\emptyset_{loss,VMD}$	Heat lost from the VMD cell, W
$\emptyset_{loss,C}$	Heat lost from the condenser, W
$\emptyset_{loss,CH1}$	Heat lost from the first chiller, W
$\emptyset_{loss,CH2}$	Heat lost from the second chiller, W
φ	Exergy flow, kJ/kg
$\Psi_{des}$	Exergy destruction, W
Subscript	
H1	Inlet hot water
H2	Outlet hot water
D1	Permeate vapor
D2	Distillate water
C1	Inlet cold water of the first chiller
C2	Outlet cold water of the first chiller
C3	Inlet cold water of the second chiller
C4	Outlet cold water of the second chiller
H	Heater
VMD	Vacuum membrane distillation
C	Condenser
CH1	First chiller
CH2	Second chiller
